# Ventilation impairment of residents around a cement plant

**DOI:** 10.1186/s40557-014-0048-6

**Published:** 2015-01-24

**Authors:** Sul Ha Kim, Chul Gab Lee, Han Soo Song, Hyun Seung Lee, Min Soo Jung, Jae Yoon Kim, Choong Hee Park, Seung Chul Ahn, Seung Do Yu

**Affiliations:** Department of Occupational & Environmental Medicine, School of Medicine, Chosun University, 558 Pilmun-daero, Dong-gu, Gwangju 501-759 Korea; National Institute of Environmental Research, 42 Hwangyong-ro, Seogu, Incheon 404-708 Korea

**Keywords:** Cement, Particulate, Pulmonary function test, Restrictive ventilation impairment

## Abstract

**Objectives:**

To identify adverse health effects due to air pollution derived from a cement plant in Korea. The ventilation impairment in residents around a cement plant was compared to another group through a pulmonary function test (PFT).

**Methods:**

From June to August of 2013, both a pre and post-bronchodilator PFT was conducted on a “more exposed group (MEG)” which consisted of 318 people who lived within a 1 km radius of a cement plant and a “less exposed group (LEG)” which consisted of 129 people who lived more than 5 km away from the same plant. The largest forced expiratory volume in a one second (FEV1) reading and a functional residual capacity (FVC) reading were recorded after examining the data from all of the usable curves that were agreed upon as valid by PFT experts of committee of National Institute of Environmental Research. The global initiative for chronic obstructive lung disease (GOLD) criteria for COPD, defined the FEV1/FVC ratio < 0.7 as the obstructive type, and the FEV1/FVC ratio ≧ 0.7 and FVC% predicted < 80% were as the restrictive type. The FVC% predicted value was estimated using Korean equation. We compared the proportion of lung function impairments between the MEG and the LEG by using a chi-square, and estimated the OR of obstructive and restrictive ventilation impairments by logistic regression.

**Results:**

The obstructive type impairment proportion was 9.7% in the MEG, whereas it was 8.5% in the LEG. The restrictive type was 21.6% in the MEG which was more than the 12.4% of the LEG. The odds ratio (OR) of total ventilation impairment in the MEG was 2.63 (95% CI 1.50 ~ 4.61) compared to the LEG. The OR of obstructive type in the MEG was 1.60 (95% CI 0.70 ~ 3.65), the smoking history was 3.10 (CI 1.10 ~ 8.66) whereas OR of restrictive type in the MEG was 2.55 (95% CI 1.37 ~ 4.76), the smoking history was 0.75 (95% CI 0.35 ~ 1.60) after adjusting for sex and age. Level of exposure to particulate played a role in both types. However, it appeared to be a significant variable in restrictive type, while smoking history was also an important variable in obstructive type.

**Conclusion:**

Although this study is a limited cross-section study with a small number of subjects, ventilation impairment rate is higher in the MEG. There might be a possibility that it is due to long-term exposure to particulate dust generated by the cement plant.

## Introduction

Although cement is the most widely used essential construction material, there are many hazardous environmental pollutants such as particulate matter, various oxides, and heavy metals, which are released in its production process. Most of the Portland cement plants in Korea have been in operation since the 1970s by the national policy to accelerate industrialization. The residents who have lived near the cement plants have always been complaining because of the inevitable dust created at plants when they were carrying limestone from the mine to the plant. However, because the protest movements from the residents have continued, epidemiological surveys have been conducted by the Ministry of Environment (National Institute of Environmental Research) since 2007 [1]. This survey was carried out in 2013, as one of a series of epidemiological investigations about the relationship between environmental particulates derived from a cement plant and pulmonary functions of the residents in Jeollanam-do Jangseong-gun.

Portland cement is composed of a mixture of materials. It is mostly made of calcium oxide (CaO, 62-67%) and silica glass (SiO_2_,17-25%) with a lesser amount of aluminum trioxide(Al_2_O_3_ 3-8%), iron oxide(Fe_2_O_3_ 0-5%), magnesium oxide (MgO, 1-2%), and other heavy metals such as hexavalent chromium (Cr^6+^), nickel, etc. [2,3]. The cement manufacturing is done in three simplified steps. First, the raw material and fuel supply preparation (mining and/or outsourcing, crushing, storage, and preblending), second the pyroprocessing to make clinker, grinding of clinker and gypsum in the finishing mill to make cement, and finally the storage, packaging, and loading for the finished products [2]. Cement is made from the intermediate product of finely ground clinker formed through a high-temperature burning of limestone and other materials in a kiln (pyroprocessing). The emissions created by the combustion such as particulates, carbon dioxide (CO_2_), nitrogen oxides, sulfur oxides, and heavy metals are discharged in the process [4]. There is additional dust generated from the mining and transportation. Various types of particulate emissions and dust are continually created by the comminution circuits when crushing and grinding the raw materials and clinker, from the pyroprocessing or kiln line, intermittently and diffusely from quarrying activities as well as through limestone transportation.

In general, fugitive emissions from coarse particulates (particularly of particle diameters >10 μm) are considered to be the reason for repeated protests by the residents near the cement plant. This is believed to be due to the visible accumulation rather than the health hazards. However, fine particulates, <10 μm (PM_10_) and <2.5 μm (PM_2.5_), can cause health problems, if one experiences prolonged exposure for 30 to 40 years, because of their respirable nature, and because they may contain potentially harmful concentrates of toxic metals and compounds. Cement dust can cause lung function impairment, pneumoconiosis [5], carcinoma of the lungs and larynx [6,7], and may cause inflammatory changes in the skin, and often leads to skin diseases or autoimmune diseases [8-10].

Many studies for cement factory workers on the adverse respiratory effects of cement dust exposure have been focusing on pulmonary function and symptoms, or their relationships [11-17]. But, few have researched the health effects of cement dust and asbestos [18,19], or heavy metals [20,21] derived from cement plants, thus a study on the relevance of dust and lung diseases of residents who live around these plants was rare. It is difficult to explain the relationship between environmental cement dust exposure and occurrence of a pulmonary disease due to the variety of other factors involved such as age, sex and smoking. Therefore, we are going to report about the difference in ventilation impairment between the more exposed group of residents and less exposed group of residents living around the cement plants or limestone mines in the region using the results of a health survey.

## Materials and methods

### Study subjects

The surveyed cement plant is located in a rural area near a big city, and about 13,000 residents live in this area. The epidemiological survey target population is about 900 over 40-year-olds living near the cement plant. The residents who lived within the 1 km radius of the cement plant were designated as the “more exposed group (MEG)”. The “less exposed group (LEG)” lived more than 5 km away from the cement plant in an area that was not typically in the path of incoming wind from the plant but was similar to the MEG in socioeconomic living conditions. The population of the eligible LEG was about 370 residents. The participants in the MEG and LEG were 453 (50.3%) and 153 (41.4%) respectively. Participants were given both pre and post-bronchodilator tests. The exceptions were persons considered to be too old or those having contraindicating reasons such as a recent myocardial infarction or infected lung disease. PFT were carried out within a university hospital and four experts of the committee of National Institute of Environmental Research determined whether the PFT test results were acceptable and reproducible. Therefore, among the participants, 447 cases were determined as valid and the pre and post-bronchodilator test results were used in the analysis; the MEG was 318 (/453 = 70.2%), the LEG was 129 (/153 = 84.3%) (Table 1). The proportions of persons with valid results of the pre and post bronchodilator test decreased with increasing age, in particular, the lowest of 46.9% in men over 70 years of the MEG.

**Table 1:** **Participants (pN) and valid pulmonary function test (vPFT) of subjects**

**Sex**	**Age**	**~59**			**60 ~ 69**			**70+**			**Total**		
		**pN**	**vPFT**	**%**	**pN**	**vPFT**	**%**	**pN**	**vPFT**	**%**	**pN**	**vPFT**	**%**
Female	LEG	26	22	84.6	29	23	79.3	45	39	79.3	100	84	84.0
	MEG	67	54	80.6	82	60	73.2	118	75	63.6	267	189	70.8
	Subtotal	93	76	82.6	111	83	76.3	163	114	71.5	367	273	74.4
Male	LEG	11	8	72.7	8	6	75.0	34	31	91.2	53	45	84.9
	MEG	62	55	88.7	60	44	73.3	64	30	46.9	186	129	69.4
	Subtotal	73	63	80.7	68	50	74.2	98	61	69.1	239	174	72.8
Total	LEG	37	30	78.7	37	29	77.2	79	70	85.3	153	129	84.3
	MEG	129	109	84.7	142	104	73.3	182	105	55.3	453	318	70.2
	Subtotal	166	139	81.7	179	133	75.2	261	175	70.3	606	447	73.8

%: Valid PFT number/participant number.

MEG: ‘more exposed group’ who lived within the 1 km radius of the cement plant.

LEG: ‘less exposed group’ who lived 5 km or more away from the cement plant in not related to usually wind direction.

### Pulmonary function test

The authors first explained the purpose of the epidemiological survey to participants, including the assumed respiratory health effect regarding the cement plant and the PFT method in more detail. We conducted PFT screenings at 9 ~ 11 AM daily by 10 ~ 20 people units from June to August in 2013. PFT was performed in a separate hospital pulmonary function laboratory following the guidelines of KOSHA and ATS/ERS TASK FORCE [22-24]. Before the test, a trained technician explained how the procedure would work and gave them a demonstration. If the elderly had a risk of falling due to syncope during a test, the PFT procedure was performed in a sitting position. The residents who refused or quit during the test were excluded from the participants. The PFT procedure was conducted twice for all participants, before and after the administration of a bronchodilator. After the pre-test was completed, 200 μg the salbutamol was inhaled twice at an interval of 30 seconds, using a total of 400 μg. The PFT procedure was then repeated after waiting for 15 minutes in a comfortable posture. Because the test results could vary due to the use of different spirometry equipment, a single model, the MicroQuark, Cosmed® (Italy), was used. We checked for abnormalities in the spirometry equipment after calibrating it by using a 3 L calibration syringe loaded into it. Each test was carried out after a preliminary examination. In addition, an investigation was conducted to asses for other factors that could affect pulmonary function, for example, past and present respiratory disease history, smoking history, residential and job history, use of firewood, general health status, history of drugs taking, recent major surgery and history of a heart disease.

### Data analysis

The FVC measurement is the maximal volume of air exhaled with maximally forced effort from a maximal respiration. The FEV1 is the maximal volume of air exhaled in the first second of a forced expiration from a position of full respiration. The FVC and FEV1 are measured through a series of at least three forced expiratory curves that have an acceptable beginning to the test and are free from artefacts, such as a cough [25]. We took the largest FVC and FEV1 results recorded (agreed to be valid by four PFT expert committees of National Institute of Environmental Research) after examining the data from all the usable curves of post-bronchodilator test. The valid tests were analyzed using a modification of the global initiative for chronic obstructive lung disease (GOLD) criteria for COPD [26], the FEV1/FVC ratio <0.7 was defined as the obstructive type of ventilation impairment, and the FEV1/FVC ratio ≧0.7 and FVC% predicted <80% was the restrictive type. We used a formula of the FVC% predicted based on Korean standard [27] (Male,-4.8434-0.00008633*age^2^(year) + 0.05292*Height (cm) + 0.01095*Weight (kg); Female, −3.0006-0.0001273*age^2^(year) + 0.03951*Height (cm) + 0.006892*Weight (kg).

Then we calculated proportions of ventilation impairment respectively in the MEG and LEG based on sex, age, residency period, current job, smoking or past occupational dust exposure history, and firewood use history. The residency period was divided into less than and more than 25 years living since the dust collection facilities of the cement plant had been incorporated in the mid-1980s. The smoking history was divided into two groups (never, current or ex-smoking) because there was no significant difference in MEG and LEG, especially in women, and the rate of women with a smoking history was as low as 4.4% (ex-smoker 1.5%, current smoker 2.9%). We tested to identify if there was a difference in proportions between ventilation impairment and other variables for two groups using a chi-square test. Variables tested included sex, age, smoking history, residency period, current job, and past occupational dust exposure or firewood use history. We used logistic regression to estimate the OR of total, obstructive and restrictive subtype impairments, using ventilation impairment as a dependent variable and other factors (two groups, sex, age and smoking history) as independent variables. A p-value of less than 0.05 was regarded to be statistically significant. All statistical analyses were performed in SPSS 21.

## Results

### General characteristic of analyzed subjects

An analysis was performed on 447 residents that showing valid results of a pre and post-bronchodilator test conducted on the 606 participants. Among the 318 in the MEG, 59.4% were male and 40.6% were female. In the LEG a total of 129 participants were included, 65.1% male and 34.9% female. This was not a significant difference (Table 2). Of the LEG 54.3% were over the age of 70, which was significantly higher than the MEG, which was 33.0%. Those with a smoking history were 33.0% and 31.8% respectively; there was no significant difference between the two groups. Similarly, there was no difference in the residence period. In regards to current employment, farmers and laborers made up 56.6% of the LEG, in contrast retired or tradesmen made up 65.7% of the MEG, which was considered to be a significant difference. The rate of using firewood was higher in the MEG. In other words, both groups were similar in sex, residence period, occupational dust exposure history, and smoking rate except for age distribution.

**Table 2:** **General characteristic of analyzed subjects**

**Variables**		**LEG (n = 29)**	**MEG (n=318)**	**Total (n = 447)**	**p-value**
Sex	Female	84 (65.1)	189 (59.4)	273 (61.1)	0.264
	Male	45 (34.9)	129 (40.6)	174 (38.9)	
Age (yrs)	≤59	30 (23.3)	109 (34.3)	139 (31.1)	0.000
	60 ~ 69	29 (22.5)	104 (32.7)	133 (29.8)	
	≥70	70 (54.3)	105 (33.0)	175 (39.1)	
Residence	≤25	29 (22.5)	84 (26.4)	113 (25.3)	0.404
	>25	100 (77.5)	234 (73.6)	334 (74.7)	
Current job	None/Merchant	56 (43.4)	209 (65.7)	265 (59.3)	0.000
	Farmer/Labor	73 (56.6)	109 (34.3)	182 (40.7)	
Smoking history	Never	88 (68.2)	213 (67.0)	301 (67.3)	0.801
	Current/ex smoking*	41 (31.8)	105 (33.0)	146 (32.7)	
Dust exposure	No	111 (86.0)	246 (77.4)	357 (79.9)	0.038
	Yes	18 (14.0)	72 (22.6)	90 (20.1)	
Firewood	No	37 (28.7)	176 (55.3)	213 (47.7)	0.000
	Yes	92 (71.3)	142 (44.7)	234 (52.3)	

p-value by Chi-square test, MEG: more exposed group, LEG: less exposed group.

*The rate of ex-smoking history in women was 1.5% (LEG 3.6%, MEG 0.5%) and current smokers were 2.9% (LEG 0.5%, MEG 3.6%).

### Ventilation impairment rate of MEG and LEG

**Figure 1:**
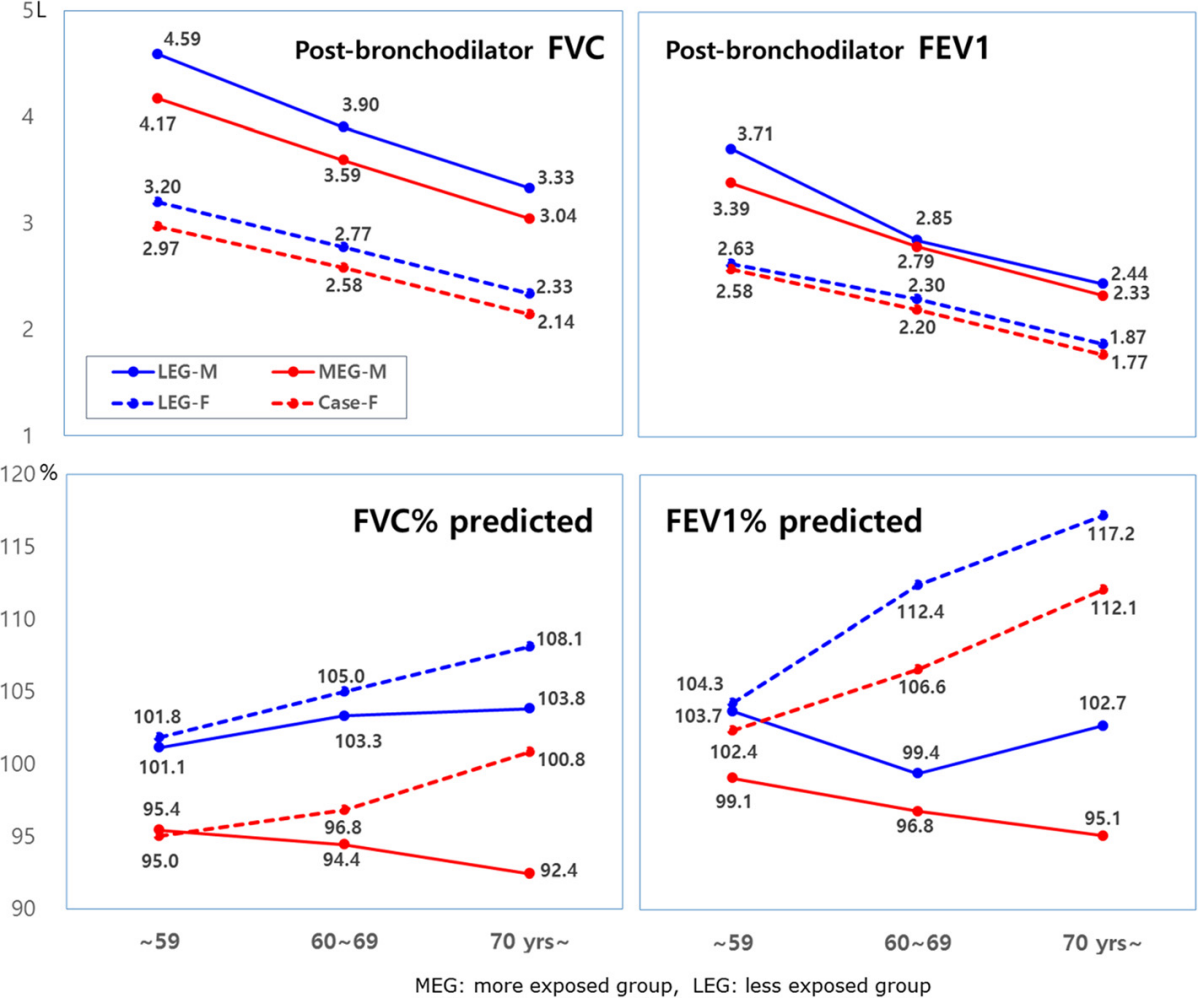
**Post-bronchodilator FVC, FEV1 (L) and predicted FVC%, FEV1%.**

In both men and women in all age groups, the post-bronchodilator mean FVC value in the MEG was significantly smaller than that of the LEG. But, the FEV1 showed no significant difference except in the ≤59 age group of men (Figure 1).

The obstructive type (FEV1/FVC <0.7) was 9.7% in the MEG, and 8.5% in the LEG. The restrictive type (FEV1/FVC ≥0.7 & FVC% predicted <80%) was 21.6% in the MEG which was higher than 12.4% of the LEG (Table 3). The ventilation impairment proportion, which is the sum of obstructive and restrictive type, was 31.3% in MEG, which was significantly higher than the 20.9% of the LEG. The proportion of ventilation impairment was higher in men than in women and also higher in older age groups and groups with a smoking history (current and ex-smoker). There was no significant difference in the ventilation impairment rate according to residency period, current job, past dust exposure related to occupational history, or firewood use history between the MEG and LEG (Table 3).

**Table 3:** **Ventilation impairment of post-bronchodilator PFT**

**Variable**		**Number**	**Ventilation impairment**	**Type**		**p-value**
				**Obstructive**	**Restrictive**	
Group	LEG	129	27 (20.9)	11 (8.5)	16 (12.4)	0.025
	MEG	318	100 (31.3)	31 (9.7)	69 (21.6)	
Sex	Female	273	52 (19.0)	8 (2.9)	44 (16.1)	0.000
	Male	174	75 (43.1)	34 (19.5)	41 (23.6)	
Age (yrs)	≤59	139	20 (14.4)	6 (4.3)	14 (10.1)	0.000
	60 ~ 69	133	37 (27.8)	13.(9.8)	24 (18.0)	
	≥70	175	70 (40.0)	23 (13.1)	47 (26.9)	
Residence period (yrs)	≤25	113	30 (26.5)	12(10.6)	18 (15.9)	0.632
	>25	334	97 (29.1)	30 (9.0)	67 (20.1)	
Occupation	None/Merchant	265	80 (30.2)	17 (6.4)	63 (23.8)	0.338
	Farmer/Labor	182	47 (25.8)	25 (13.7)	22 (12.1)	
Smoking history	Never	301	65 (21.6)	11 (3.7)	54 (17.9)	0.000
	Current/ex smoking	146	62 (42.4)	31 (21.2)	31 (21.2)	
Dust exposure occupational history	No	357	97 (27.2)	31 (8.7)	66 (18.5)	0.295
	Yes	90	30 (33.3)	11 (12.2)	19 (21.1)	
Firewood use history	No	213	60 (28.2)	20 (9.4)	40 (18.8)	0.917
	Yes	234	67 (28.6)	22 (9.4)	45 (19.2)	

p-value on total ventilation impairment by Chi-square test, MEG: more exposed group, LEG: less exposed group.

Ventilation impairment: Obstructive type: FEV1/FVC <0.7, Restrictive type: FEV1/FVC ≥0.7 & FVC% predicted <80%.

The OR value for each variable was adjusted by enter method of logistic regression (Table 4). The OR of the ventilation impairment rate in the MEG was 2.63 (95% CI 1.50 ~ 4.61) compared to the LEG. The OR in male was 3.30 (95% CI 1.68 ~ 6.48), higher than that of female. The OR according to age, in 60–69 and ≧70 years was 2.92 (95% CI 1.53-5.56), 7.03 (95% CI 3.71-13.32) compared with <59 years respectively. OR of ventilation impairment rate in the smoking history was 1.44 (95% CI 0.72 ~ 2.84) compared to the non-smoker. But, OR of obstructive type in the MEG was 1.60 (95% CI 0.70 ~ 3.65), the smoking history was 3.10 (CI 1.10 ~ 8.66) whereas OR of restrictive type in the MEG was 2.55 (95% CI 1.37 ~ 4.76), the smoking history was 0.75 (95% CI 0.35 ~ 1.60). Statistical significance was adjusted depending on the obstructive or restrictive type of ventilation impairment. In restrictive type, level of exposure to particulate was a significant variable while smoking history was also an important variable in obstructive type.

**Table 4:** **Adjusted odds ratio and CI of ventilation impairments**

		**Ventilation impairment**		**Obstructive type**		**Restrictive type**	
Group	LEG	1					
	MEG	2.63	(1.50-4.61)	1.60	(0.72-3.65)	2.55	(1.37-4.76)
Sex	Female	1					
	Male	3.30	(1.68-6.48)	4.16	(1.35-12.83)	2.26	(1.09-4.66)
Age (yrs)	≤59	1					
	60 ~ 69	2.92	(1.53-5.60)	3.20	(1.13-9.06)	2.09	(1.01-4.29)
	≥70	7.03	(3.71-13.32)	5.74	(2.09-15.76)	4.34	(2.20-8.54)
Smoking history	Never	1					
	Current/ex smoking	1.44	(0.72-2.84)	3.10	(1.10-8.66)	0.75	(0.35-1.60)

Logistic regression with adjusted for all variables, CI: 95% confidence interval, MEG: more exposed group, LEG: less exposed group.

Ventilation impairment, Obstructive type; FEV1/FVC <0.7, Restrictive type; FEV1/FVC ≥0.7 & FVC% predicted <80%.

## Discussion

The residents who live around the cement plant have always complained about suffering in their daily lives because of dust originating from the plant. These complaints included respiratory symptoms such as chronic cough or phlegm as well as itching sensations from the body surface and prickly feeling eyes. While the residents refer to the irritant as ‘cement dust’ it should be noted that ‘particulate’ is the more scientific term. A particulate is anything solid or liquid suspended in the air [28]. It not only includes primary particles coming from limestone powder or directly out of the exhaust from a kiln during preprocessing, but also can include secondary particles, such as sulfates and nitrates, which are formed during the condensation of vaporized materials or from the by-products of the oxidation of gases in the atmosphere. Among these particles, particulates (<10 μm, PM_10_) and fine particulates (<2.5 μm, PM_2.5_) that were derived intermittently and diffusely from raw materials and other manufactured products appear to be more of a health hazard because of their respirable nature and because they may contain potentially harmful concentrations of toxic metals and compounds [4,29]. Various gases are generated directly from clinker manufacturing, according to a recent study on the health effects of fine particulates and how they affect mortality [30].

In order to determine the health effects of particulates, pulmonary function tests have been used for quite a long time. Ventilation impairments in cement plant workers is already well documented [3,11-15]. According to relatively consistent reports, the FVC and FEV1 of such workers is significantly decreased when compared to their matched control groups. These were also meaningfully decreased based on the length employment [3]. However, the foundation of these studies is different than the situation affecting the health of the residents. These studies reported that the concentration of respirable dust in the workplace for 8 h/day shift ranged from 3.7 mg/m^3^ (kilns) to 23 mg/m^3^ (ore crushing area) [8]. It was the highest for the crusher at 27.49 mg/m^3^, 16.90 mg/m^3^ around the packing areas and 1.55 mg/m^3^ in administration offices throughout the production process [14,15,17].

**Figure 2:**
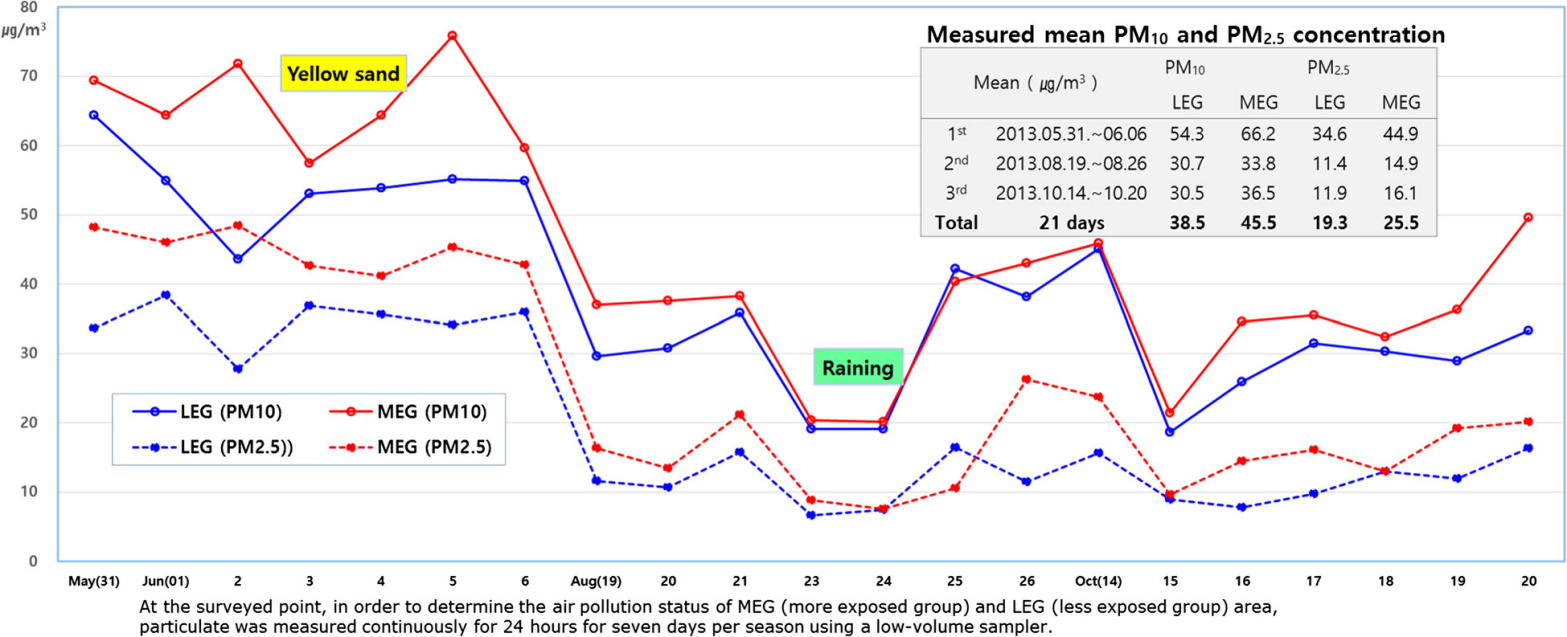
**Difference of measured PM**_**10**_ **and PM**_**2.5**_ **concentration in a cement plant area.**

**Figure 3:**
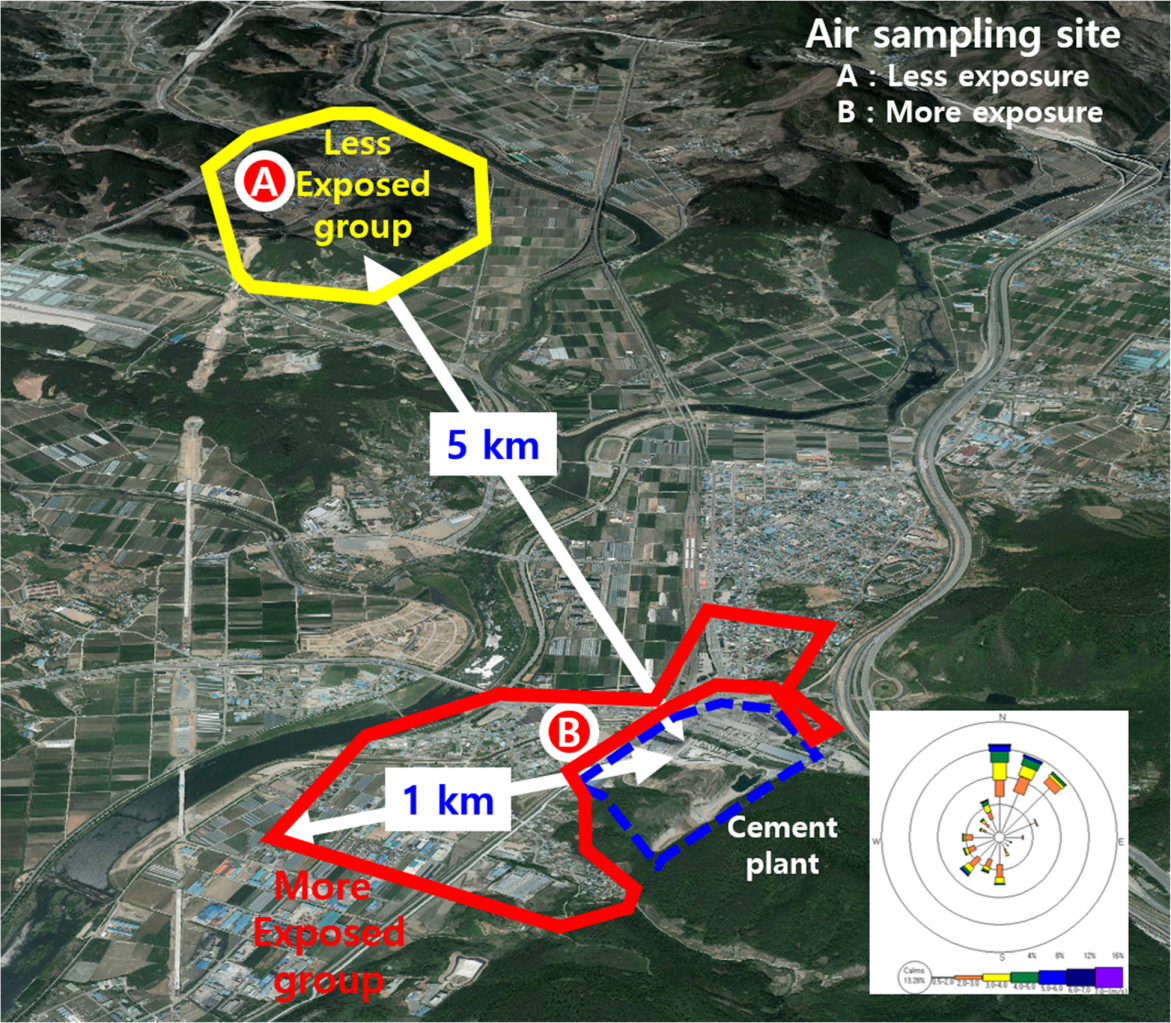
**MEG and LEG position.**

However, it seems that there is a significant difference found in health effects between the studies examining ambient pollution and in studies about ventilation impairment among cement plant laborers. This is likely to be due to the concentration of dust within the plant being much higher than the outside of the plant. How is it possible to confirm that the health effects are the result of prolonged exposure to particulate arising from a cement plant? It’s a question of whether the respiratory health effects of residents with ventilation impairments found using PFT can be proved to be due to the low concentration long-term exposure to particulates from the cement plant. This study used the data of particulate concentrations collected in a total of 21 days in June, August and October. Data was collected every seven days. The mean PM_10_ concentration in the atmosphere was 45.5 μg/m^3^ (95% CI 37.8 ~ 53.3) beside the cement plant, higher than 38.5 μg/m^3^ (95% CI 32.3 ~ 44.7) in a 5 km away point from the cement plant. Also, the mean PM_2.5_ concentration was 25.5 μg/m^3^ (95% CI 18.7 ~ 32.3), higher than 19.3 μg/m^3^ (95% CI 14.1 ~ 24.6), respectively (Figure 2). Of course, this does not measure the level of exposure of the past and may only reflects the current situation. However, it can be assumed that residents who live close to the cement plant were exposed to higher levels of particulate before dust collection facilities had been implemented in mid-1980s. When the plant location is used as the central location on the map (Figure 3), the LEG lived more than 5 km away in the northwest and the MEG lived within 1 km in the southwest. The wind direction is a northwester during the three seasons in the fall, winter and spring, and is a southwester during summer. The LEG habitats were in an independent wind direction while the MEG was right on the side of the plant, where the northwestern wind blows during three seasons, having the plant in the southwest of the area. It was confirmed that the particulate concentration in cement plant area (MEG region) was higher than that in the control area (LEG region) as well as continuously being exposed to more particulates due to the wind direction. Even so, the concentration around cement plant area would still be lower than the inside of the cement plant.

This study had obvious limitations that included the very small number of surveyed population as well as being a cross-sectional study. Moreover, the pulmonary function test performs a fundamental role in clinical setting, but its result varies with sex, age, height, and ethnicity. Furthermore, ventilation impairment degrees are greatly affected by smoking, occupational exposure (including organic or inorganic dusts), chemical agents and fumes [26], and the use of firewood as cooking or heating fuel in poorly ventilated kitchens. The smoking rates in Korean men were close to 80% in the 1980 ~ 90s [31]. Firewood has been used for a long time including recently as both a cooking and heating fuel source in the rural regions of Korea. Also, the target population in this study was residents who lived near the cement plant, and because many of them are current farmers or had farmed, they were always exposed to dust in the soil and to chemical substances like agriculture chemicals. In spite of these limitations, we were trying to assess pulmonary function status of the MEG who lived near the cement plant compared with the LEG who was living under similar economic living conditions and in sociocultural environments, for example, the use of firewood as cooking or heating fuel, similar types of occupations and smoking rates.

Everyone who was over the age of 40 living in these areas was to receive the pulmonary function test and we also encouraged them to participate actively. As a result, about 50% of the expected target population was examined. In validation checks on pre and post bronchodilator test, 26.2% of the participants were eliminated because the test reproducibility did not meet criteria [25] when matched with three acceptable curves or due to early termination of expiration. Therefore, approximately 40% of the target population was analyzed. The proportion of valid PTF readings in the MEG was 70.2%, but it was 84.2% in the LEG. Despite the PFT tests being carried out under the same conditions in a stable hospital room, there was difference in invalid test proportions between the two groups (Table 1). Unfortunately, the authors also could not find out why there was a significant difference in valid PFT proportions between the two groups. However, if the proportion of valid PFT proportions between the two groups had been similar, then it would mean that there would be a higher obstructive and restrictive ventilation impairment in the MEG while it would be lower in the LEG.

The classification of ventilation impairment is based on a modified GOLD criterion for COPD definition [26,32]. Regardless of the presence of symptoms like chronic coughing or sputum, and dyspnea, the obstructive type was considered to be FEV1/FVC <0.7 in post-bronchodilator readings. If the FVC% predicted was less than 80%, it was considered the restrictive type, even if the FEV1/FVC was above of 0.7. Although the PFT tests showed about 15% difference in invalid test proportions between the two groups, it put us on the spot to compare the two groups for ventilation impairment. Whatever results came from the analysis, the most characteristic finding was a 10% difference in the restrictive type between two groups. There was less visible difference in obstructive type, 9.7% in the MEG and 8.5% in the LEG (Table 3). The relationship between particulates and COPD or increasing mortality rate in respiratory disease is well known [29,33]. The prevalence of COPD was 14.0% in those with the post-bronchodilator test, whereas the prevalence was 20.9% in those with a pre-bronchodilator test during 2007–2010, the National Health and Nutrition Examination Survey of Americans aged 40–79 years [34]. Similarly, the prevalence of COPD in post-bronchodilator tests ranged from 23.4% to 11.6% in 10,360 adults aged 40 years and older in 14 countries in North America, Europe, Africa and Asia who participated in the Burden of Obstructive Lung Disease study [35]. The prevalence rates in China and the Philippines, which belong to Asia like Korea, were the lowest at 11.6% and 12.7%, respectively. When compared with such reports, a prevalence of obstructive type of ventilation impairment in this study was presumed to be similar.

By the way, how can we say that restrictive ventilation impairment in the MEG is much higher than that of LEG? In the regression analysis, the OR of all ventilation impairment was higher in persons of older age and who are men. Smoking is known to be the biggest cause of COPD (chronic obstructive pulmonary disease) [26,36]. We have not separated the number of ex and current smokers in Table 2, because there was no significant difference in both groups. In men in MEG, 37.2% were ex-smokers and 39.5% were current smokers, whereas in men in LEG, 48.9% and 28.9%, respectively. In women, the percentages were very low. In women of MEG, 0.5% were ex-smokers and 2.6% were current smokers, whereas in LEG, 3.6% were ex-smokers and 3.6% were current smokers. Therefore, the reason the obstructive type OR of men against women in Table 4 is as high as 4.16 (95% CI 1.35 ~ 12.83) is because most of the ex and current smokers are men. In fact, the MEG and LEG are similar people living in the same rural area with similar economic and socio-cultural settings. The aim of this study is to determine whether the particles from the cement plant have an effect on ventilation impairment, so we did not need to subdivide the smoking history into more categories.

Restrictive impairment was unrelated to smoking history, while smoking history was a main risk factor of for obstructive impairment. Restrictive impairment may occur because of increased lung recoil caused by pulmonary fibrotic change or weakened respiratory systems due to old age, or both [37]. Particulates were in the lung parenchyma, blood vessel walls, airway, lymphoid follicles and alveolar macrophages [33]. Particulates can be sensed by the airway’s epithelial cells, activate macrophages, dendritic cells and innate immune cells. They can then initiate responses in various populations of specific immune cells such as T helper cells, T cytotoxic cells and B cells. Initiation of inflammatory immune responses, activation of immune cells and release of many cytokines, chemokines and other inflammatory molecules, have variable pathologic effects like fibrotic change [38]. There is a limit which is that we could not confirm the total lung capacity (TLC) for restrictive type impairment by body plethysmography or DLco (diffusion capacity of lung for carbon monoxide) [39], in the few residents without a history of occupational dust-related who were diagnosed with pneumoconiosis.

The strength of this study is that at present a particulate (PM 10 and PM 2.5) concentration was measured to evaluate a level of dust exposure related a cement plant; the concentration of the particulate was higher in the area around the cement plant (MEG) than the other area (LEG). And we have compared the health effects by chronic particulate exposure according to GOLD’s guideline (post-bronchodilator spirometry) [40]. The PFT was carried out in order to obtain a reliable value by the same examiner in comfortable and stable environment in the hospital. However, we need to take the limitations into account when interpreting the result. This is a cross-sectional study with not enough number of residents to find a significant difference in the ventilation impairment, especially in the obstructive type between the two groups. ﻿We have reviewed other data related to this epidemiologic survey such as population change, the lung cancer incidence and mortality due to respiratory diseases, 70% of the residents who were living in the area when the cement plant was first built (1973) have moved to larger cities (as of 2013). And there is also a possibility that the residents whose health had been compromised have already died of lower respiratory tract disorder or lung cancer. The residents who were too old to go through PFT or have been diagnosed with contraindicated diseases have been excluded from the study. In addition, there were significantly lower valid PFT proportions in MEG (70.2%) compared to those of LEG (84.3%). Therefore, it is possible that the health effects of chronic particulate exposure has are actually underestimated.

## Conclusion

Although this study was a limited cross-section study with a small number of subjects, the ventilation impairment rate, particularly in restrictive not obstructive type, is higher in the MEG than the LEG even with a lower valid PFT proportion in MEG. There might be a possibility that it is due to long-term exposure to particulate dust generated from the cement plant.

## References

[1] Leem JH, Cho JH, Lee EC, Kim JH, Lee DH, Lee SJ, Lee JY, Kim HC (2010). Clusters of pneumoconiosis among residents near cement factories. Korean J Occup Environ Med.

[2] Van Oss H, Padovani AC (2002). Cement manufacture and the environment: part I: chemistry and technology. J Ind Ecol.

[3] Meo SA (2004). Health hazards of cement dust. Saudi Med J.

[4] Van Oss H, Padovani AC (2003). Cement manufacture and the environment: part II: environmental challenges and opportunities. J Ind Ecol.

[5] Abrons HL, Petersen MR, Sanderson WT, Engelberg AL, Harber P (1997). Chest radiography in Portland cement workers. J Occup Environ Med.

[6] Smailyte G, Kurtinaitis J, Andersen A (2004). Mortality and cancer incidence among Lithuanian cement producing workers. Occup Environ Med.

[7] Dietz A, Ramroth H, Urban T, Ahrens W, Becher H (2004). Exposure to cement dust, related occupational groups and laryngeal cancer risk: results of a population based case‐control study. Int J Cancer.

[8] Goon ATJ, Goh CL (2000). Epidemiology of occupational skin disease in Singapore 1989–1998. Contact Dermatitis.

[9] Spoo J, Elsner P (2001). Cement burns: a review 1960–2000. Contact Dermatitis.

[10] Wang B, Wu J-D, Sheu S-C, Shih T-S, Chang H-Y, Guo Y-L, Wang Y-J, Chou T-C (2011). Occupational hand dermatitis among cement workers in Taiwan. J Formos Med Assoc.

[11] Al-Neaimi Y, Gomes J, Lloyd O (2001). Respiratory illnesses and ventilatory function among workers at a cement factory in a rapidly developing country. Occup Med.

[12] Merenu I, Mojiminiyi F, Njoku C, Ibrahim M (2007). The effect of chronic cement dust exposure on lung function of cement factory workers in sokoto, Nigeria. Afr J Biomed Res.

[13] Mirzaee R, Kebriaei A, Hashemi S, Sadeghi M, Shahrakipour M (2008). Effects of exposure to Portland cement dust on lung function in Portland cement factory workers in Khash, Iran. IJEHSE.

[14] Poornajaf A, Kakooei H, Hosseini M, Ferasati F, Kakaei H (2010). The effect of cement dust on the lung function in a cement factory, Iran. IJOH.

[15] Zeleke ZK, Moen BE, Bratveit M (2010). Cement dust exposure and acute lung function: a cross shift study. BMC Pulm Med.

[16] Nordby K-C, Fell AKM, Notø H, Eduard W, Skogstad M, Thomassen Y, Bergamaschi A, Kongerud J, Kjuus H (2011). Exposure to thoracic dust, airway symptoms and lung function in cement production workers. Eur Respir J.

[17] Ahmed HO, Abdullah AA (2012). Dust exposure and respiratory symptoms among cement factory workers in the United Arab Emirates. Ind Health.

[18] Kumagai S, Kurumatani N (2009). Asbestos fiber concentration in the area surrounding a former asbestos cement plant and excess mesothelioma deaths in residents. Am J Ind Med.

[19] Musti M, Pollice A, Cavone D, Dragonieri S, Bilancia M (2009). The relationship between malignant mesothelioma and an asbestos cement plant environmental risk: a spatial case–control study in the city of Bari (Italy). Int Arch Occup Environ Health.

[20] Gbadebo A, Bankole O (2007). Analysis of potentially toxic metals in airborne cement dust around Sagamu, southwestern Nigeria. J Appl Sci.

[21] Cha KT, Oh SS, Yoon JH, Lee KH, Kim SK, Cha BS, Kim SH, Eom AY, Koh SB (2011). Adverse health outcomes in residents exposed to cement dust. J Toxicol Environ Health Sci.

[22] KOSHA (2013). The technical guideline on pulmonary function test and interpretation KOSHA GUIDE.

[23] FORCE AET, Miller MR, Crapo R, Hankinson J, Brusasco V, Burgos F, Casaburi R, Coates A, Enright P, van der Grinten CM, Gustafsson P (2005). General considerations for lung function testing. Eur Respir J.

[24] Townsend MC (2011). Spirometry in the occupational health setting-2011 update. J Occup Environ Med.

[25] FORCE AET, Miller MR, Hankinson J, Brusasco V, Burgos F, Casaburi R, Coates A, Crapo R, Enright P, Van Der Grinten C, Gustafsson P (2005). Standardisation of spirometry. Eur Respir J.

[26] Vestbo J, Hurd SS, Agusti AG, Jones PW, Vogelmeier C, Anzueto A, Barnes PJ, Fabbri LM, Martinez FJ, Nishimura M (2013). Global strategy for the diagnosis, management, and prevention of chronic obstructive pulmonary disease: GOLD executive summary. Am J Respir Crit Care Med.

[27] Choi JK, Paek D, Lee JO (2005). Normal predictive values of spirometry in Korean population. Tuberc Respir Dis (Seoul).

[28] Dockery DW (2009). Health effects of particulate air pollution. Ann Epidemiol.

[29] Ling SH, van Eeden SF (2009). Particulate matter air pollution exposure: role in the development and exacerbation of chronic obstructive pulmonary disease. Int J Chron Obstruct Pulmon Dis.

[30] Burnett RT, Pope CA, Ezzati M, Olives C, Lim SS, Mehta S, Shin HH, Singh G, Hubbell B, Brauer M, Anderson HR, Smith KR, Balmes JR, Bruce NG, Kan H, Laden F, Pruss-Ustun A, Turner MC, Gapstur SM, Diver WR, Cohen A (2014). An integrated risk function for estimating the global burden of disease attributable to ambient fine particulate matter exposure. Environ Health Perspect.

[31] Khang Y-H, Cho H-J (2006). Socioeconomic inequality in cigarette smoking: trends by gender, age, and socioeconomic position in South Korea, 1989–2003. Prev Med.

[32] Booker R (2005). Best practice in the use of spirometry. Nurs Stand.

[33] Ling SH, McDonough JE, Gosselink JV, Elliott WM, Hayashi S, Hogg JC, van Eeden SF (2011). Patterns of retention of particulate matter in lung tissues of patients with COPD. Chest.

[34] Tilert T, Dillon C, Paulose-Ram R, Hnizdo E, Doney B (2013). Estimating the U.S. prevalence of chronic obstructive pulmonary disease using pre- and post-bronchodilator spirometry: the National Health and Nutrition Examination Survey (NHANES) 2007–2010. Respir Res.

[35] Tan WC, Vollmer WM, Lamprecht B, Mannino DM, Jithoo A, Nizankowska-Mogilnicka E, Mejza F, Gislason T, Burney PG, Buist AS (2012). Worldwide patterns of bronchodilator responsiveness: results from the burden of obstructive lung disease study. Thorax.

[36] Mannino DM, Buist AS (2007). Global burden of COPD: risk factors, prevalence, and future trends. The Lancet.

[37] FORCE AET, Pellegrino R, Viegi G, Brusasco V, Crapo R, Burgos F, Casaburi R, Coates A, Van der Grinten C, Gustafsson P, Hankinson J (2005). Interpretative strategies for lung function tests. Eur Respir J.

[38] Esmaeil N, Gharagozloo M, Rezaei A, Grunig G (2014). Dust events, pulmonary diseases and immune system. Am J Clin Exp Immunol.

[39] FORCE AET, Wanger J, Clausen J, Coates A, Pedersen O, Brusasco V, Burgos F, Casaburi R, Crapo R, Enright P, Van Der Grinten C (2005). Standardisation of the measurement of lung volumes. Eur Respir J.

[40] Sterk P (2004). Let’s not forget: the GOLD criteria for COPD are based on post-bronchodilator FEV1. Eur Respir J.

